# Supplementary material for: An Antibacterial Peptide with High Resistance to Trypsin Obtained by Substituting d-Amino Acids for Trypsin Cleavage Sites
Source: Antibiotics (Basel). 2021 Nov 28;10(12):1465. doi: 10.3390/antibiotics10121465 (PMC8698302; doi:10.3390/antibiotics10121465)
Supplement: Supplementary file 1 [file antibiotics-10-01465-s001.zip › antibiotics-1446132-supplementary.pdf]

Supplementary Materials

An Antibacterial Peptide with High Resistance to Trypsin Obtained by Substituting D-Amino Acids for Trypsin Cleavage Sites

Xiaoou Zhao [1,†], Mengna Zhang [1,†], Inam Muhammad [1,2], Qi Cui [1], Haipeng Zhang [3,4], Yu Jia [3,4], Qijun Xu [1], Lingcong Kong [1,*] and Hongxia Ma [3,4,*]

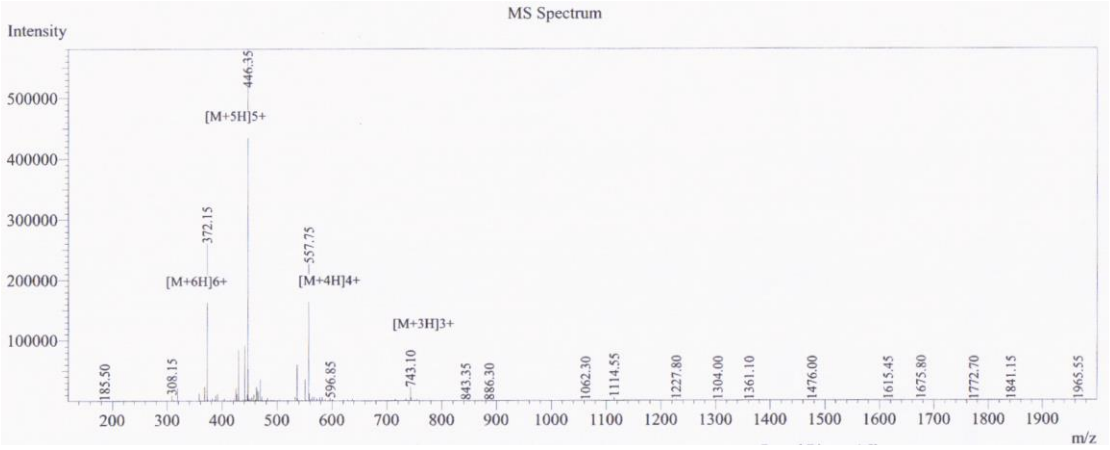

(a) VDKPPYLPRPRPIRRPGGR-NH_2(OM19R)

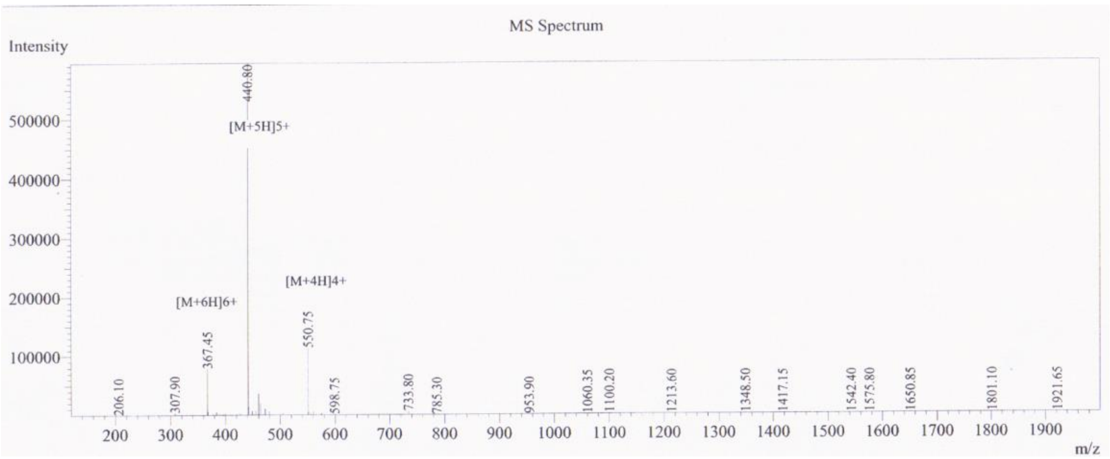

(b) ADKPPYLPRPRPIRRPGGR-NH_2

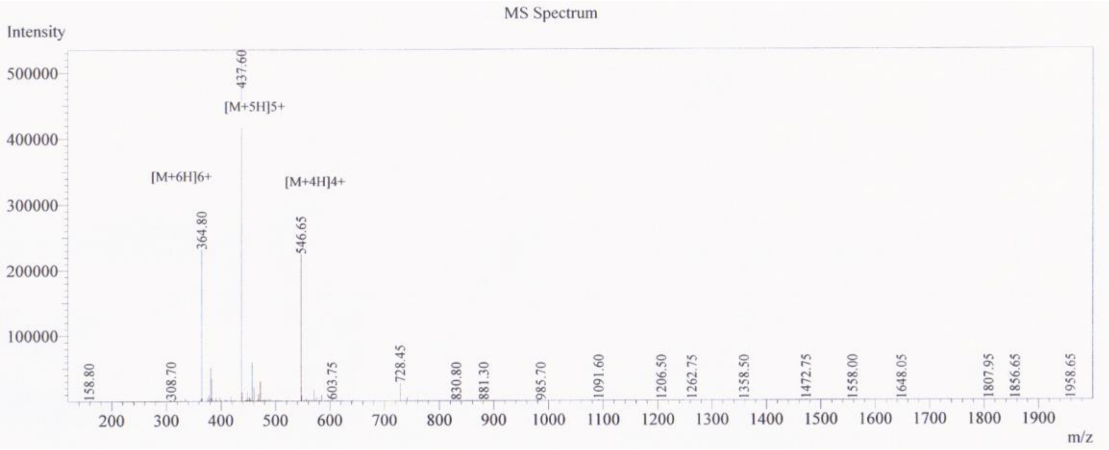

(c) VAKPPYLPRPRPIRRPGGR-NH_2

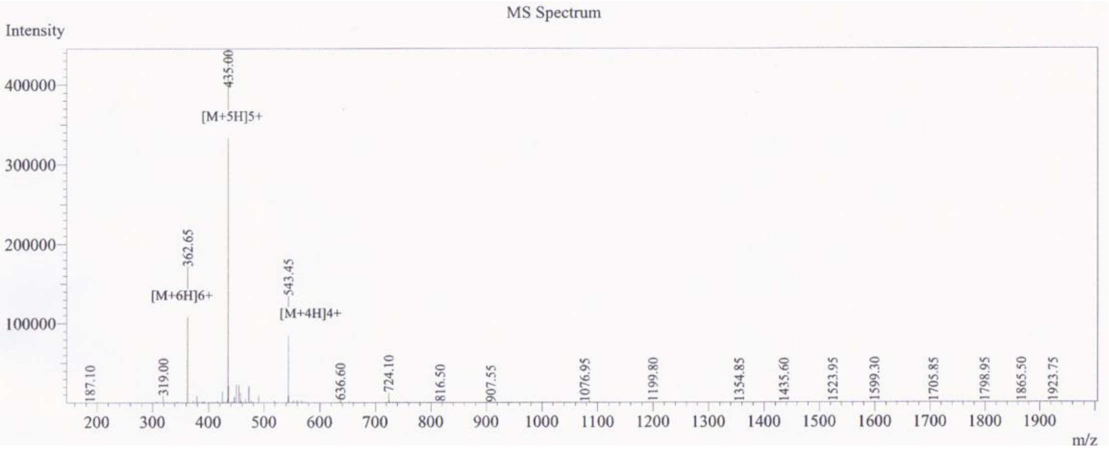

(d) VDAPPYLPRPRPIRRPGGR-NH_2

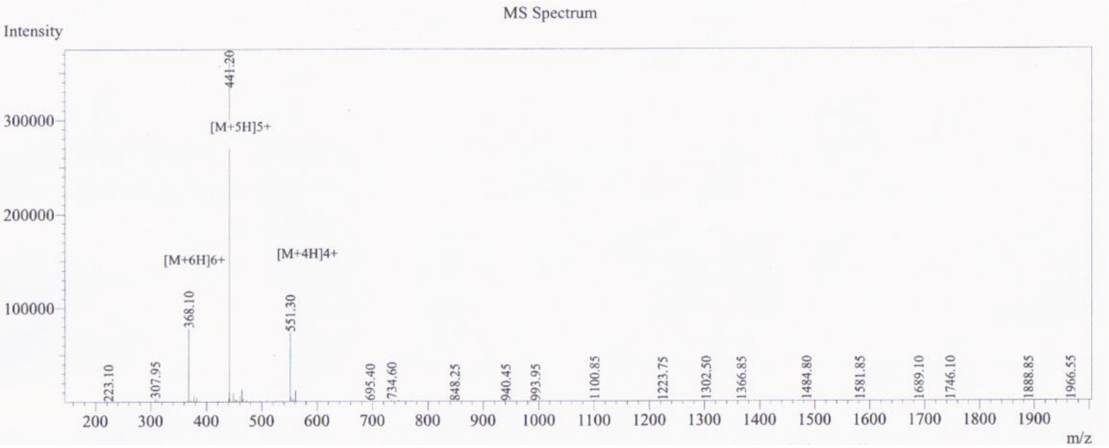

(e) VDKAPYLPRPRPIRRPGGR-NH_2

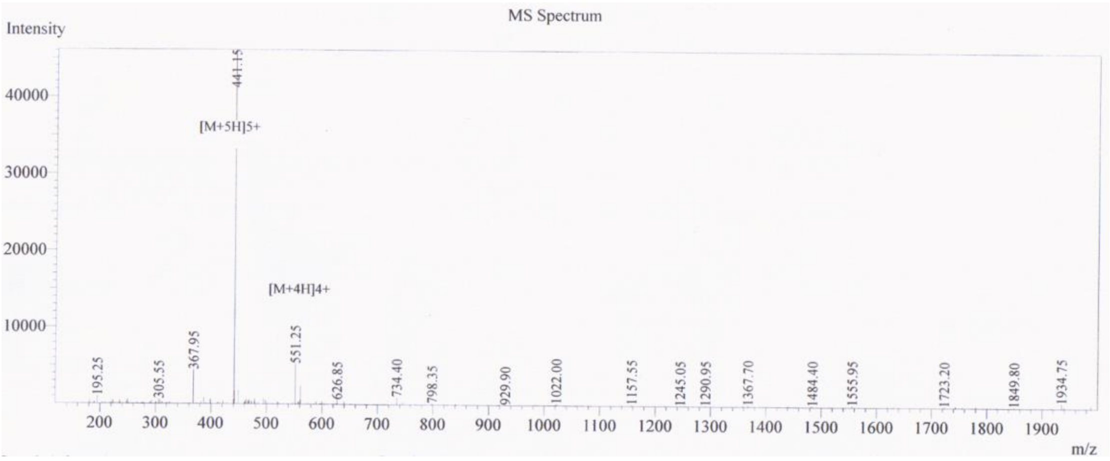

(f) VDKPAYLPRPRPIRRPGGR-NH2

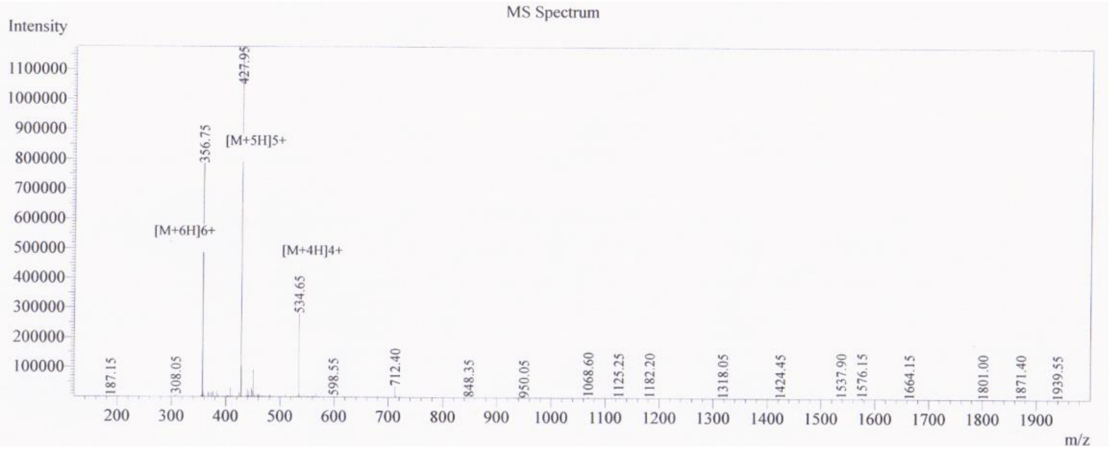

(g) VDKPPALPRPRPIRRPGGR-NH2

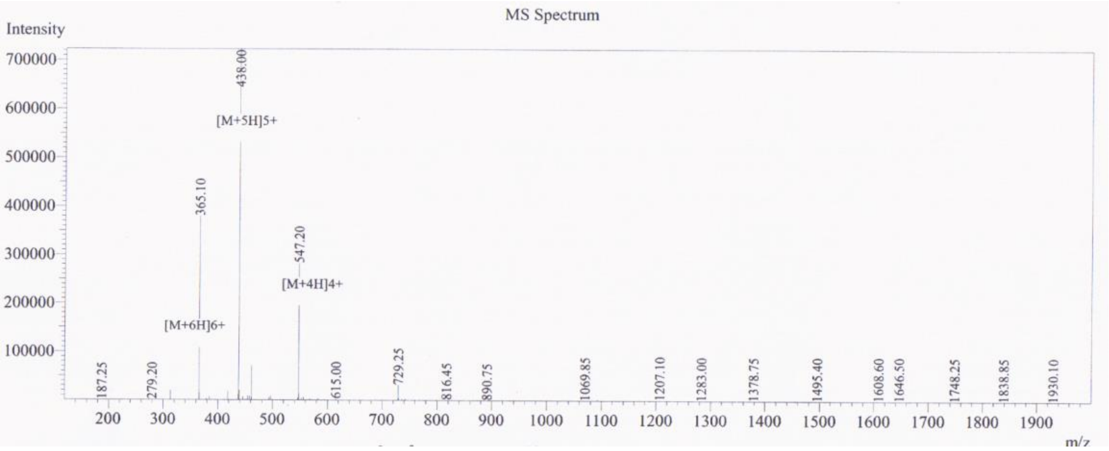

(h) VDKPPYAPRPRPIRRPGGR-NH2

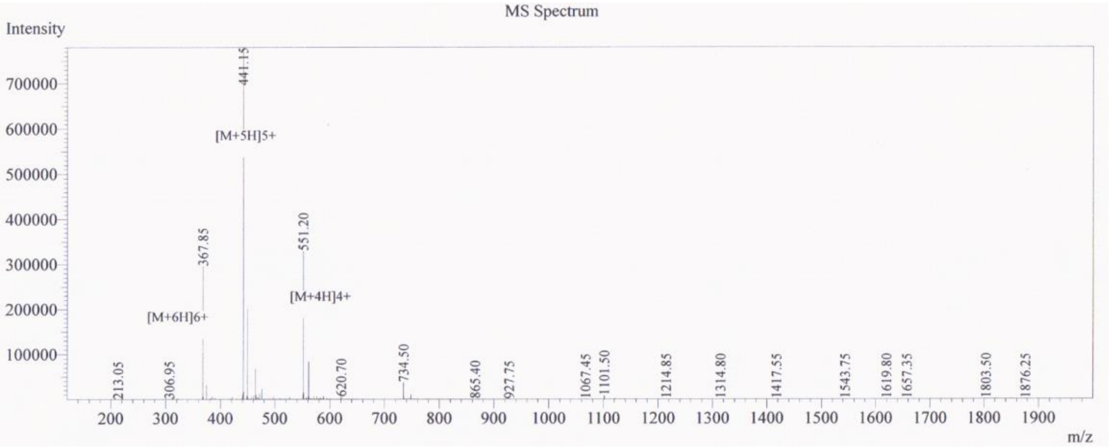

(i) VDKPPYLARPRPIRRPGGR-NH2

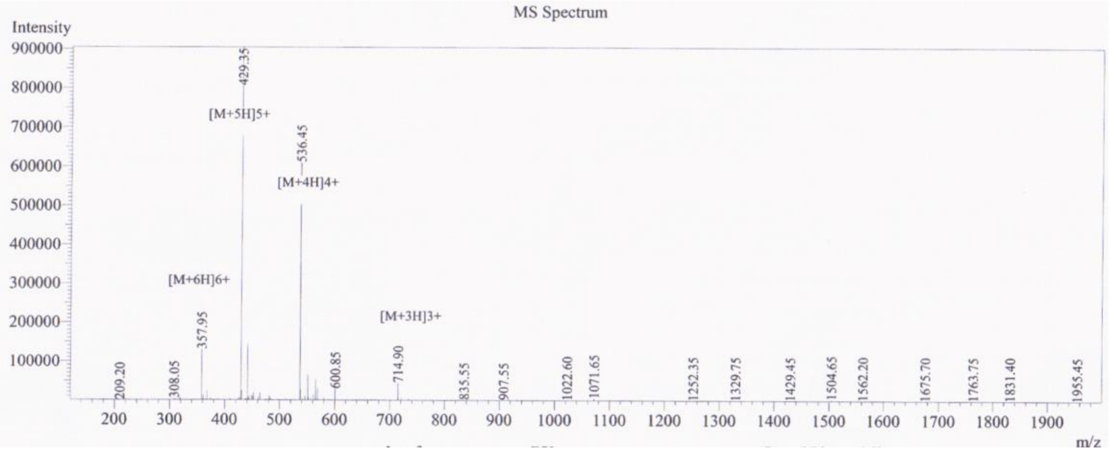

(j) VDKPPYLPAPRPIRRPGGR-NH2

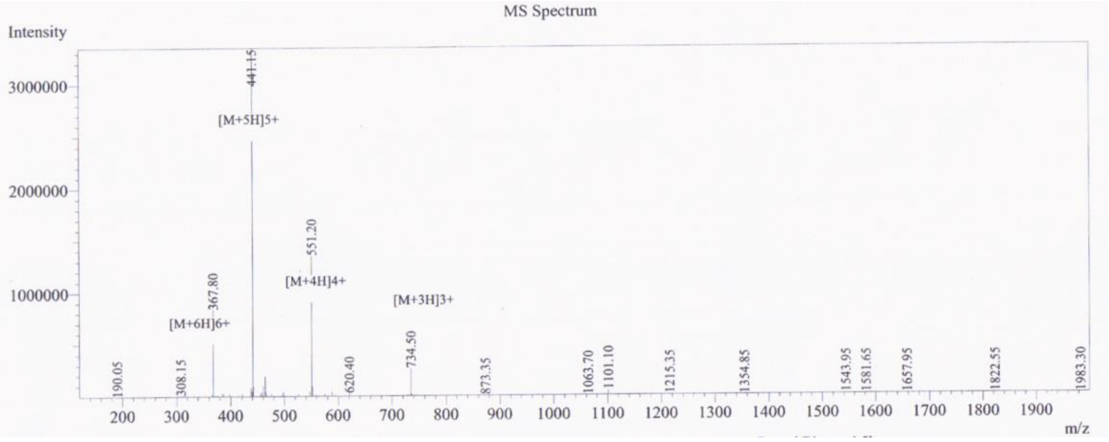

(k) VDKPPYLPRARPIRRPGGR-NH2

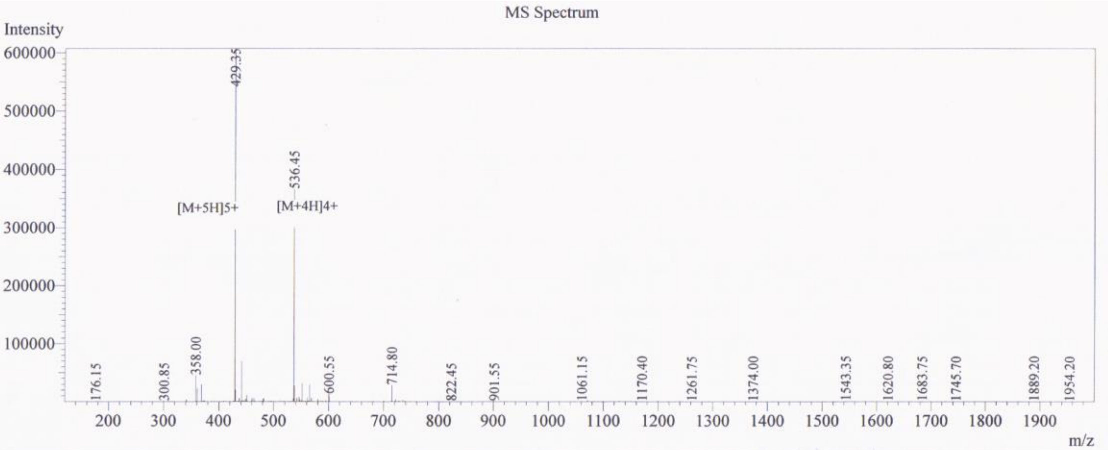

(l) VDKPPYLPRPAPIRRPGGR-NH_2

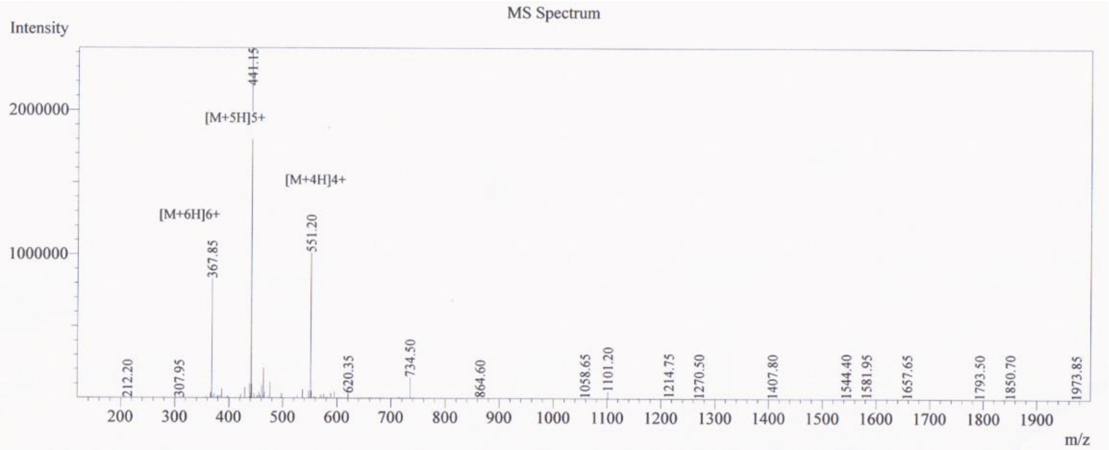

(m) VDKPPYLPRPRAIRRPGGR-NH_2

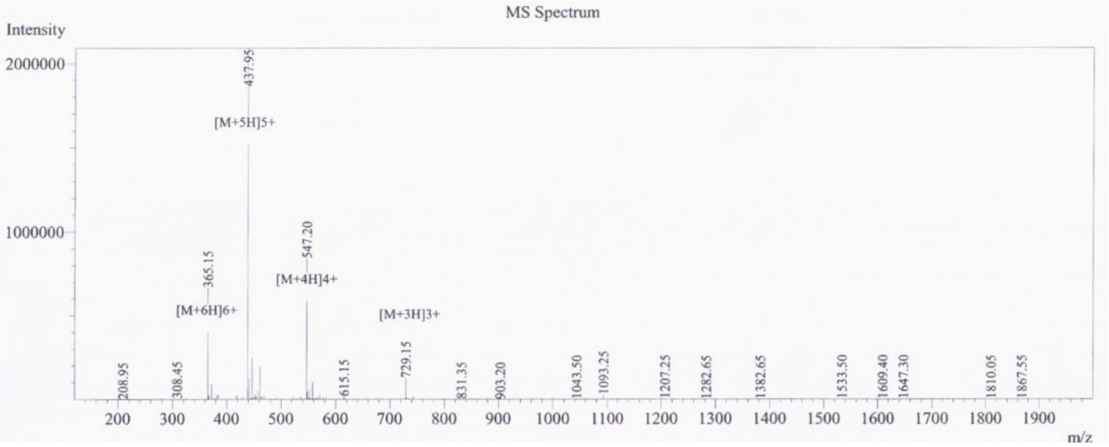

(n) VDKPPYLPRPRPARRPGGR-NH_2

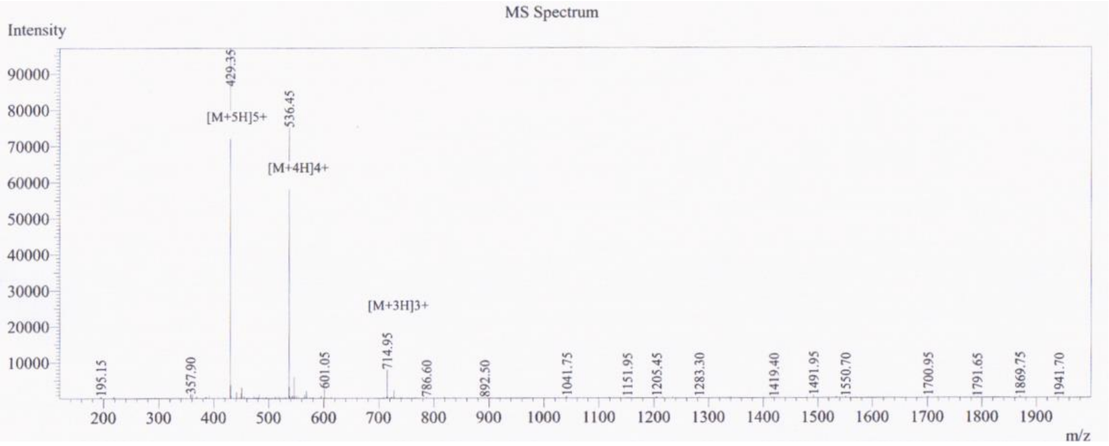

(o) VDKPPYLPRPRPIARPGGR-NH_2

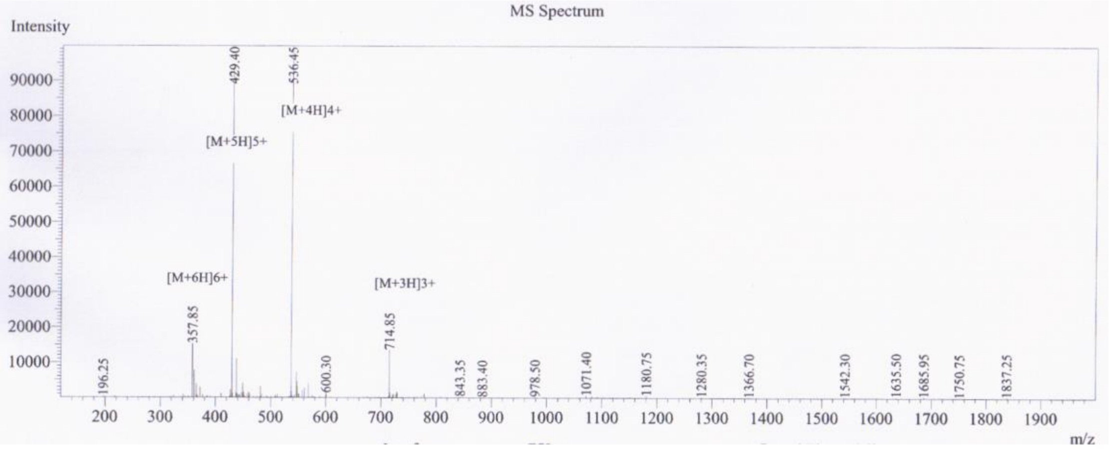

(p) VDKPPYLPRPRPIRAPGGR-NH_2

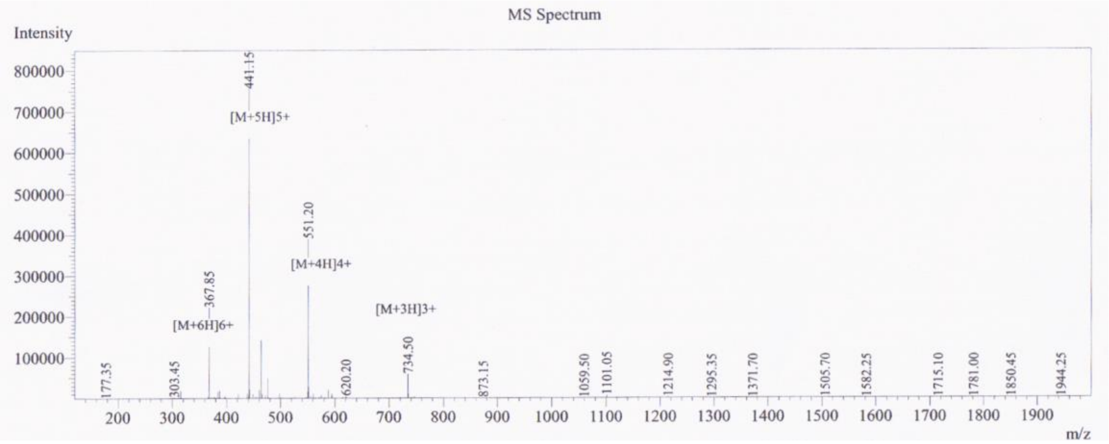

(q) VDKPPYLPRPRPIRRAGGR-NH_2

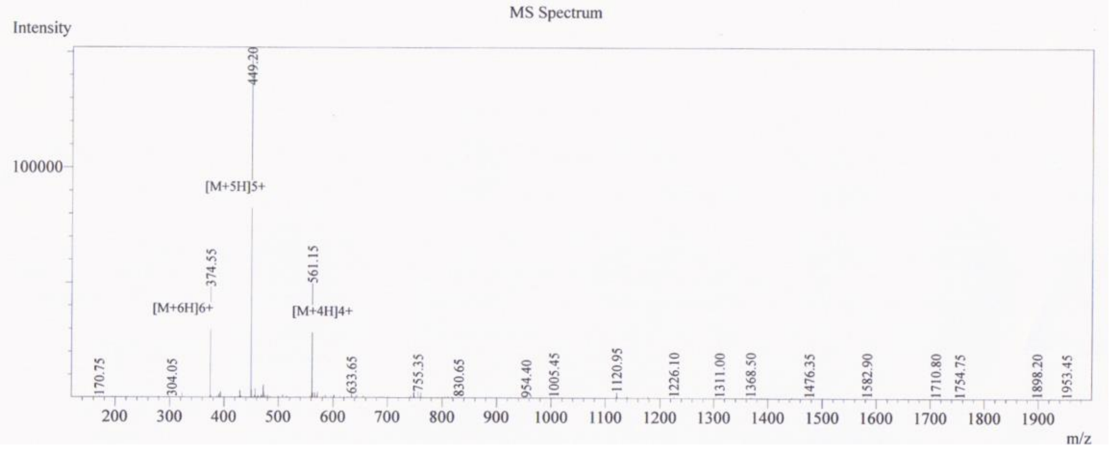

(r) VDKPPYLPRPRPIRRPAGR-NH2

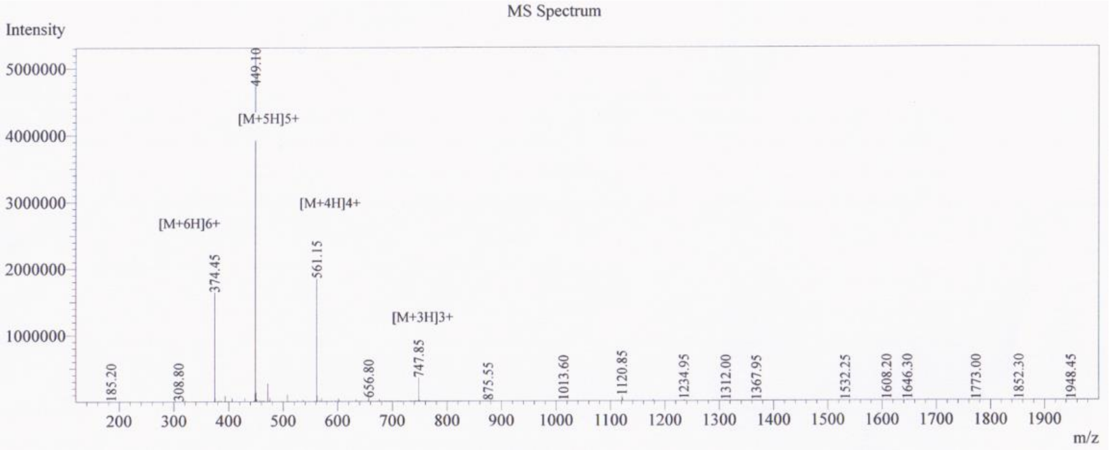

(s) VDKPPYLPRPRPIRRPGAR-NH2

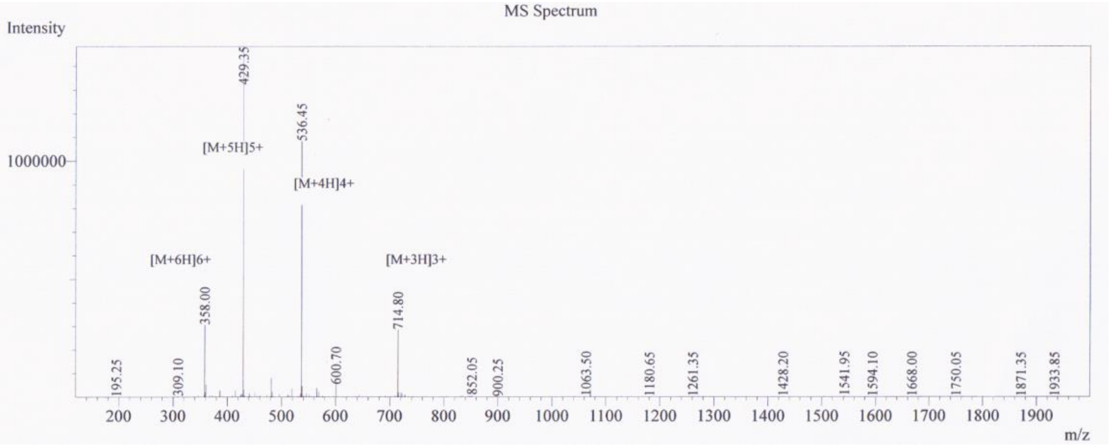

(t) VDKPPYLPRPRPIRRPGGA-NH2

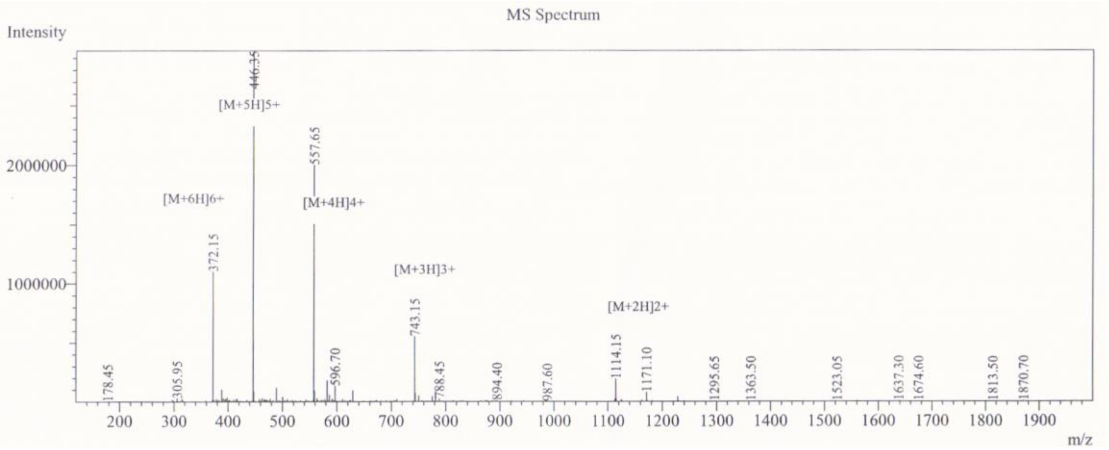

(u) VDkPPYLPrPrPIrrPGGr-NH2 (OM19D)

Figure S1. The ESI-MS of Peptides

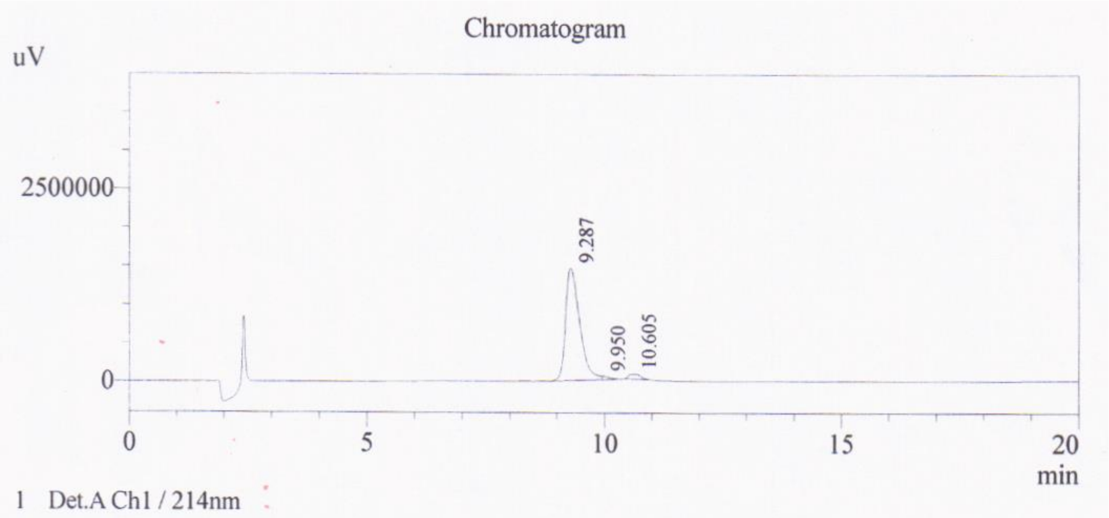

PeakTable

Detector A Ch1 214nm

Peak#	Ret. Time	Area	Height	Area %	Height %
1	9.287	30204028	1466022	95.116	93.032
2	9.950	443906	44529	1.398	2.826
3	10.605	1107158	65269	3.487	4.142
Total		31755091	1575820	100.000	100.000

(a) VDKPPYLPRPRPIRRPGGR-NH2（OM19R）

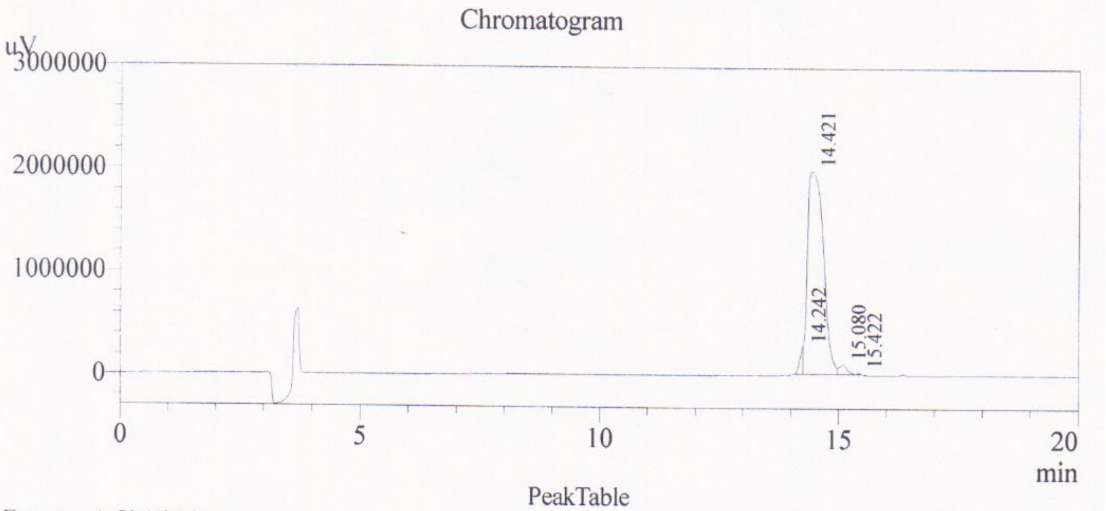

PeakTable

Detector A Ch1 214nm

Peak#	Ret. Time	Area	Height	Area %	Height %
1	14.242	1169630	254089	2.466	10.958
2	14.421	45172035	1964701	95.231	84.728
3	15.080	1030974	89995	2.173	3.881
4	15.422	61499	10058	0.130	0.434
Total		47434138	2318842	100.000	100.000

(b) ADKPPYLPRPRPIRRPGGR-NH_2

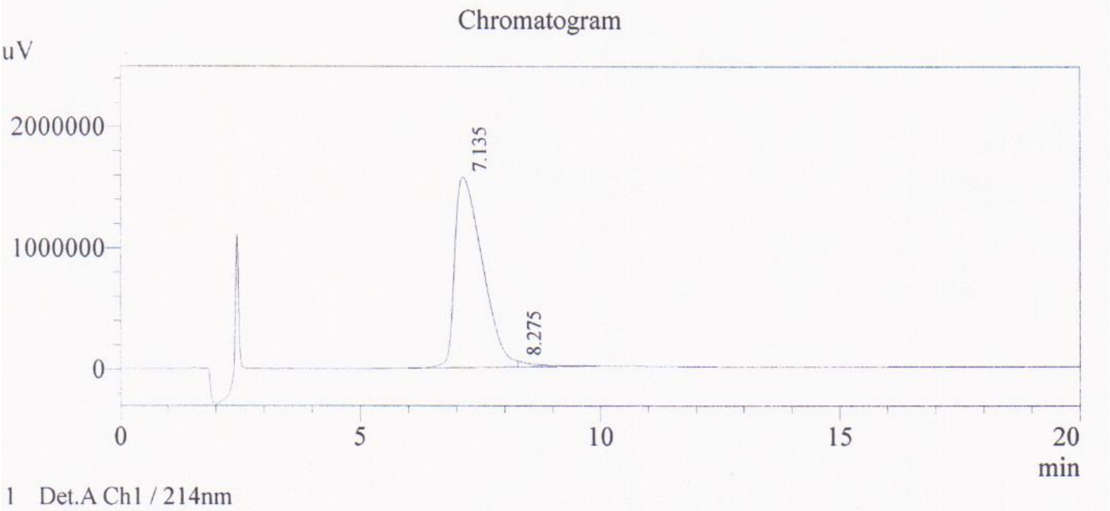

PeakTable

Detector A Ch1 214nm

Peak#	Ret. Time	Area	Height	Area %	Height %
1	7.135	62803294	1571611	98.135	96.972
2	8.275	1193437	49077	1.865	3.028
Total		63996731	1620689	100.000	100.000

(c) VAKPPYLPRPRPIRRPGGR-NH_2

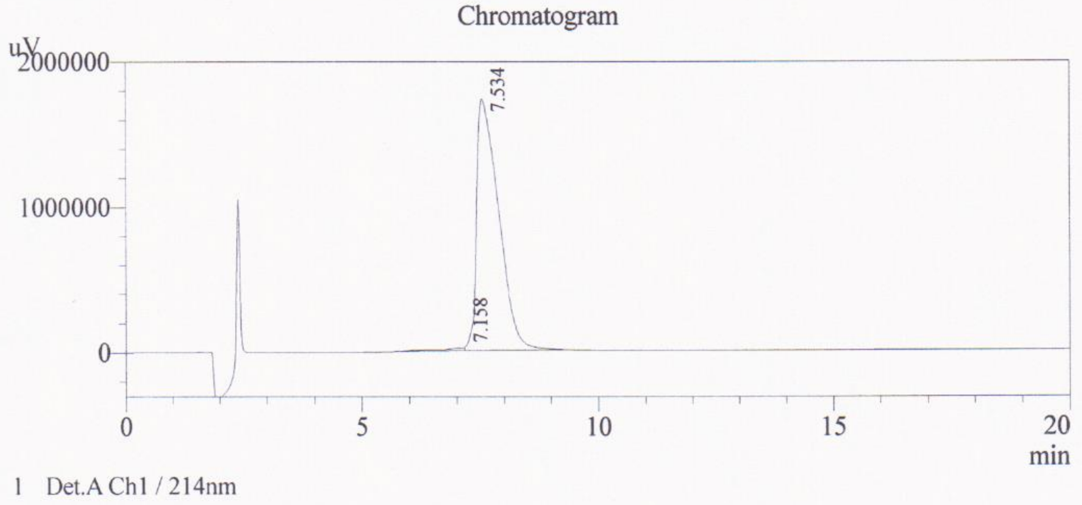

PeakTable

Detector A Ch1 214nm

Peak#	Ret. Time	Area	Height	Area %	Height %
1	7.158	692286	20059	1.219	1.147
2	7.534	56090035	1729024	98.781	98.853
Total		56782321	1749082	100.000	100.000

(d) VDAPPYLPRPRPIRRPGGR-NH_2

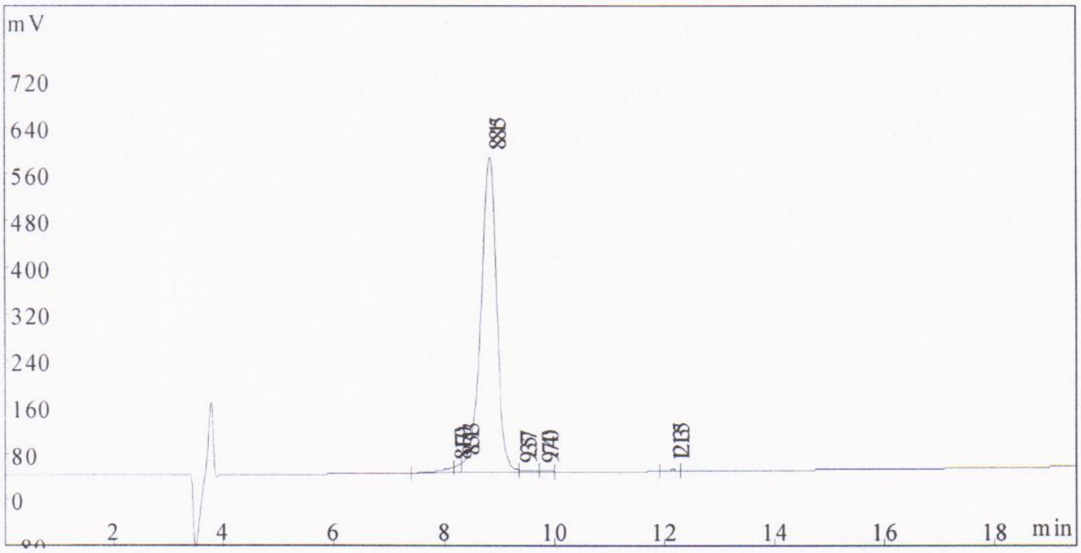

PeakTable

Detector A Ch 214nm

Peak	Ret.Time	Area	Height	Area%	Height%
1	8.170	133671	9404	1.245	1.641
2	8.313	110093	16389	1.025	2.861
3	8.815	10414410	539195	96.991	94.113
4	9.357	44526	3082	0.415	0.538
5	9.740	13906	2315	0.130	0.404
6	12.135	20799	2538	0.194	0.443
Total		10737405	572923	100.000	100.000

(e) VDKAPYLPRPRPIRRPGGR-NH_2

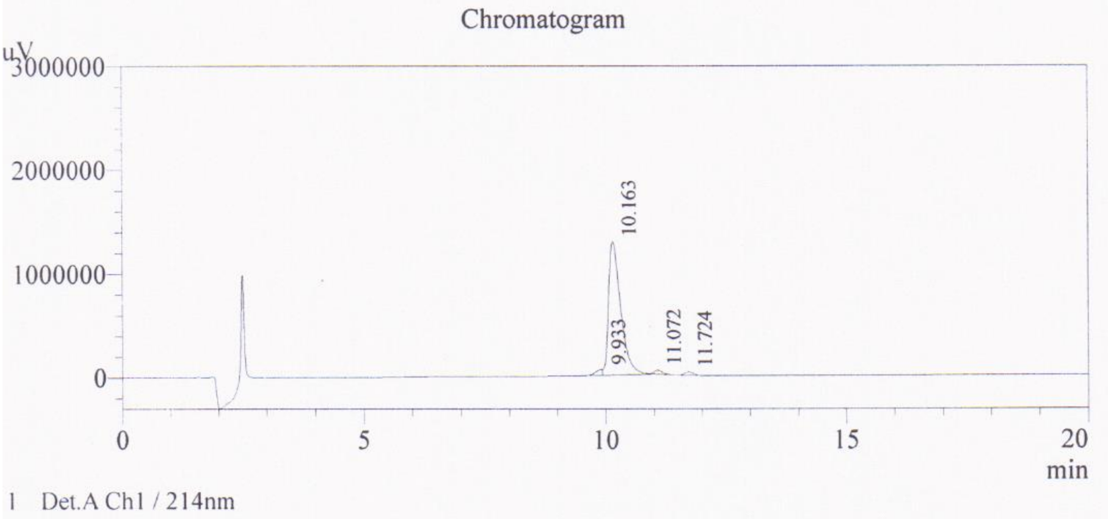

PeakTable

Detector A Ch1 214nm

Peak#	Ret. Time	Area	Height	Area %	Height %
1	9.933	460772	51307	1.964	3.655
2	10.163	22307850	1286921	95.075	91.670
3	11.072	367636	35656	1.567	2.540
4	11.724	327079	29980	1.394	2.136
Total		23463336	1403864	100.000	100.000

(f) VDKPAYLPRPRPIRRPGGR-NH_2

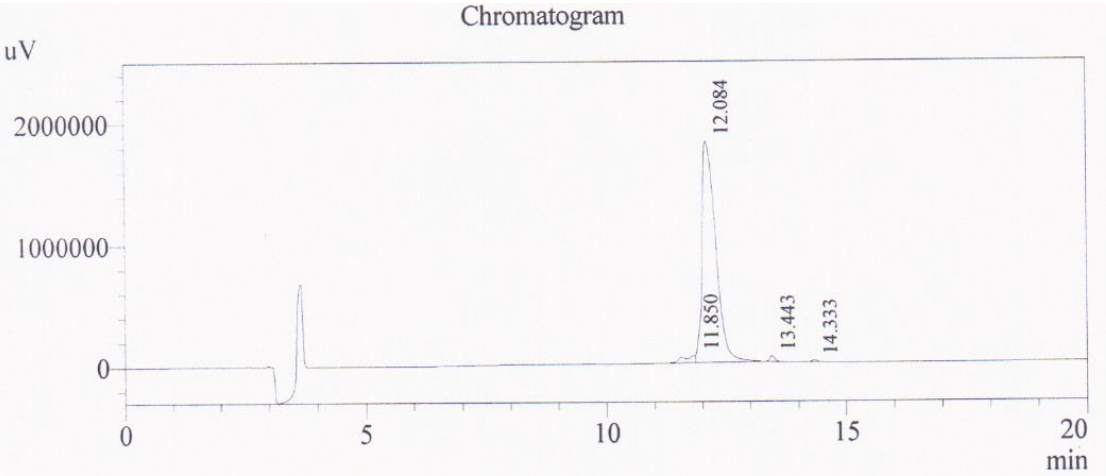

PeakTable

Detector A Ch1 214nm

Peak#	Ret. Time	Area	Height	Area %	Height %
1	11.850	1025099	61035	2.710	3.128
2	12.084	36344614	1823960	96.070	93.468
3	13.443	350542	50310	0.927	2.578
4	14.333	110937	16114	0.293	0.826
Total		37831192	1951419	100.000	100.000

(g) VDKPPALPRPRPIRRPGGR-NH_2

Chromatogram

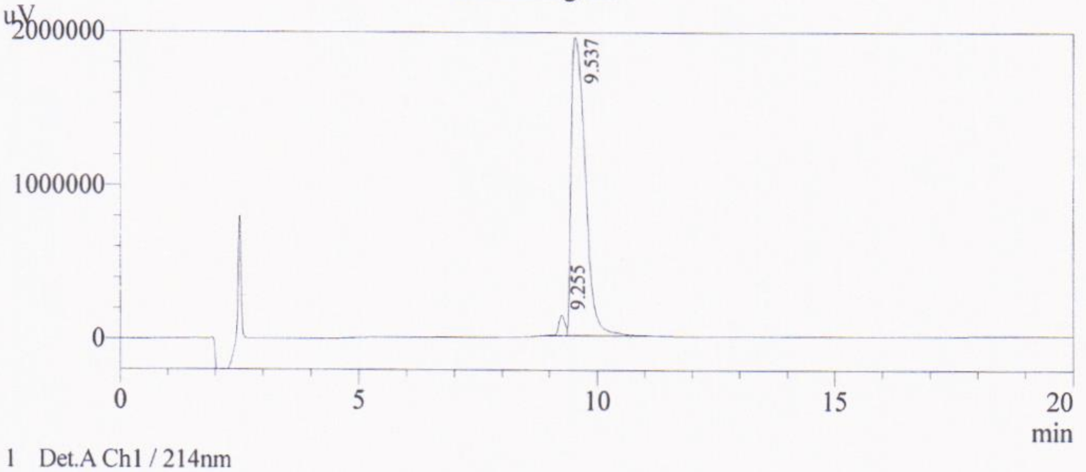

1 Det.A Ch1 / 214nm

PeakTable

Detector A Ch1 214nm

Peak#	Ret. Time	Area	Height	Area %	Height %
1	9.255	1194243	134011	2.912	6.451
2	9.537	39816066	1943238	97.088	93.549
Total		41010309	2077249	100.000	100.000

(h) VDKPPYAPRPRPIRRPGGR-NH_2

Chromatogram

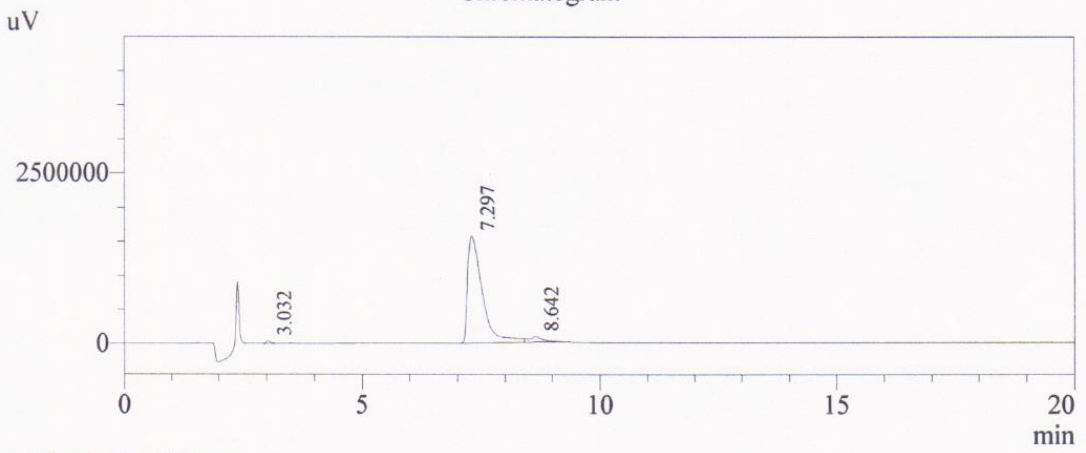

1 Det.A Ch1 / 214nm

PeakTable

Detector A Ch1 214nm

Peak#	Ret. Time	Area	Height	Area %	Height %
1	3.032	220017	34929	0.620	2.070
2	7.297	33743833	1570214	95.040	93.059
3	8.642	1541177	82185	4.341	4.871
Total		35505027	1687329	100.000	100.000

(i) VDKPPYLARPRPIRRPGGR-NH_2

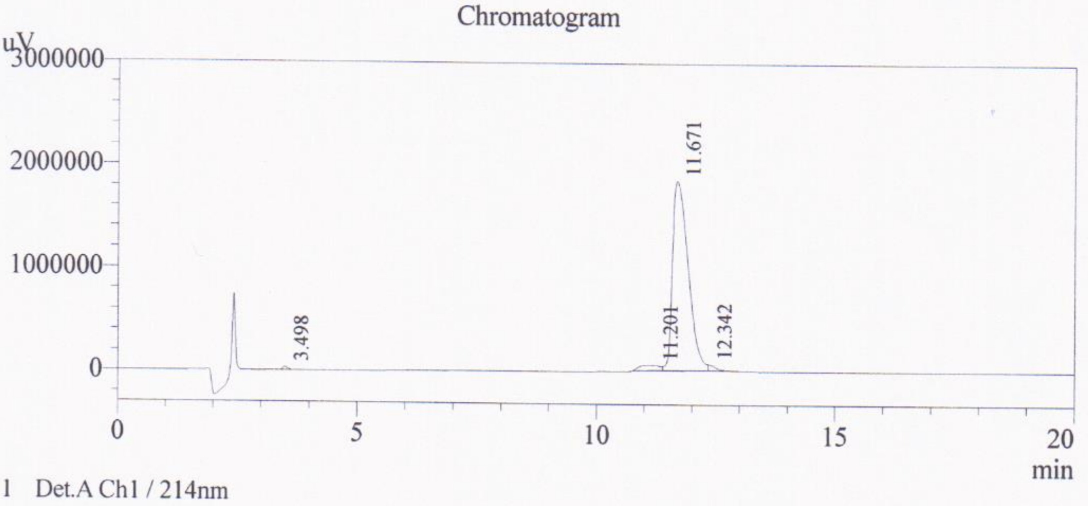

PeakTable

Detector A Ch1 214nm

Peak#	Ret. Time	Area	Height	Area %	Height %
1	3.498	212998	29682	0.473	1.504
2	11.201	1452805	49028	3.225	2.484
3	11.671	42868197	1836371	95.148	93.055
4	12.342	520174	58348	1.155	2.957
Total		45054174	1973428	100.000	100.000

(j) VDKPPYLPAPRPIRRPGGR-NH_2

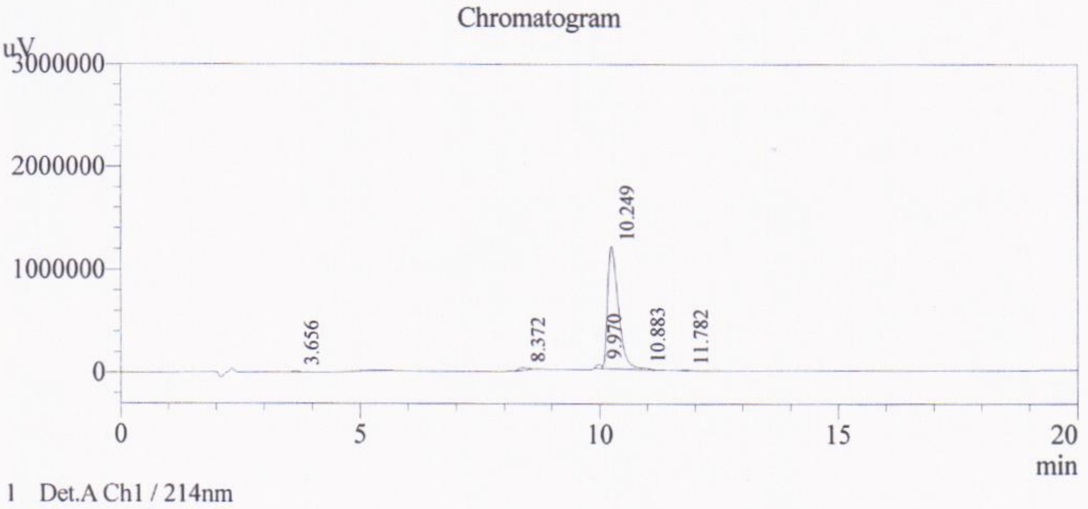

PeakTable

Detector A Ch1 214nm

Peak#	Ret. Time	Area	Height	Area %	Height %
1	3.656	96737	12590	0.494	0.975
2	8.372	302667	25626	1.547	1.985
3	9.970	292455	43629	1.495	3.380
4	10.249	18680819	1190448	95.475	92.235
5	10.883	154921	14305	0.792	1.108
6	11.782	38567	4068	0.197	0.315
Total		19566166	1290666	100.000	100.000

(k) VDKPPYLPRARPIRRPGGR-NH_2

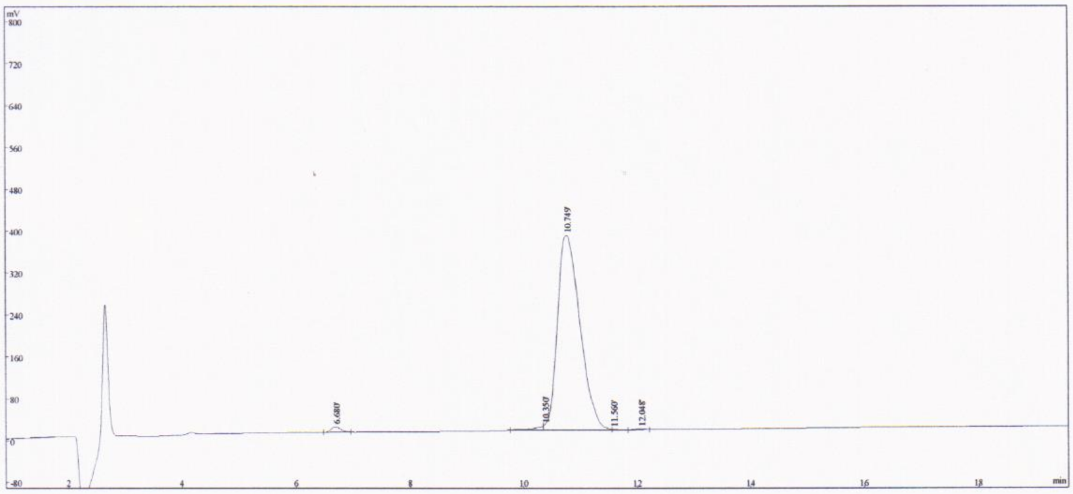

PeakTable

Detector A Ch 214nm

Peak	Ret.Time	Area	Height	Area%	Height%
1	6.680	102253	9523	1.011	2.435
2	10.350	85644	8611	0.847	2.202
3	10.749	9906048	370442	97.939	94.731
4	11.560	3243	960	0.032	0.246
5	12.048	17241	1510	0.171	0.386
Total		10114429	391046	100.000	100.000

(l) VDKPPYLPRPAPIRRPGGR-NH_2

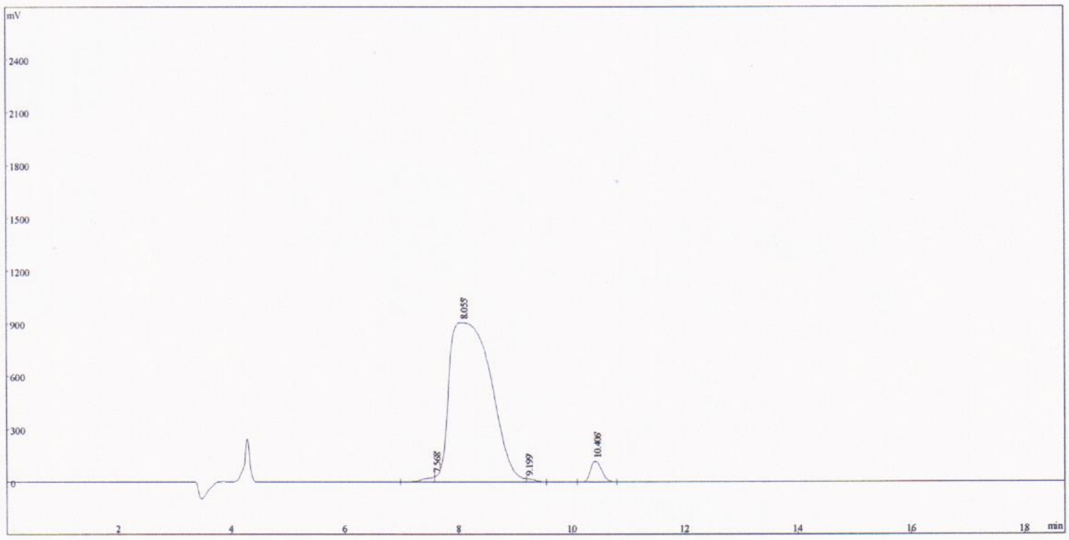

PeakTable

Detector A Ch 214nm

Peak	Ret.Time	Area	Height	Area%	Height%
1	7.568	435092	32104	0.894	2.966
2	8.055	46383222	909290	95.339	84.005
3	9.199	191822	20213	0.394	1.867
4	10.406	1641251	120819	3.373	11.162
Total		48651387	1082426	100.000	100.000

(m) VDKPPYLPRPRAIRRPGGR-NH_2

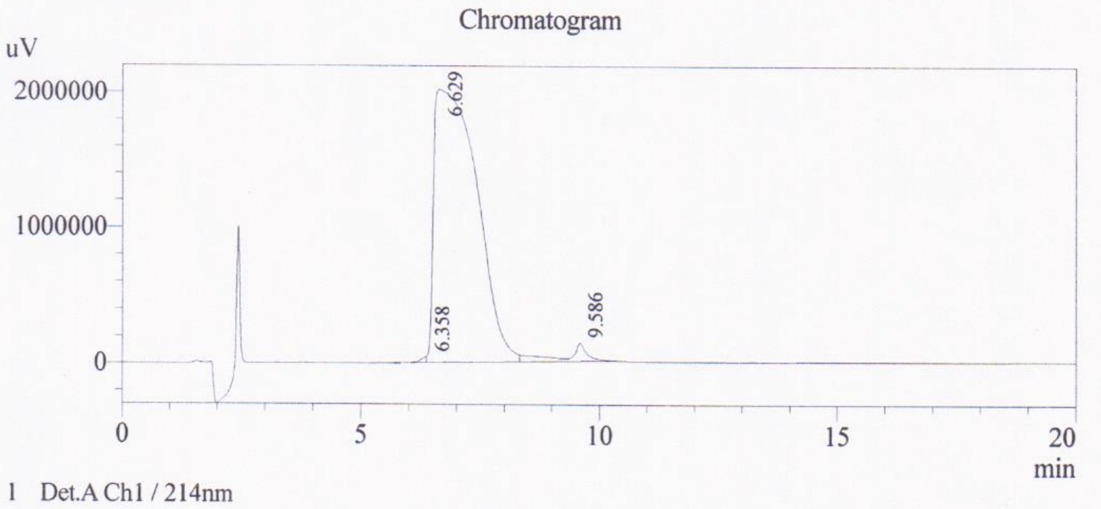

PeakTable

Detector A Ch1 214nm

Peak#	Ret. Time	Area	Height	Area %	Height %
1	6.358	338955	41969	0.259	1.912
2	6.629	126030402	2015780	96.385	91.844
3	9.586	4387470	137036	3.355	6.244
Total		130756827	2194786	100.000	100.000

(n) VDKPPYLPRPRPARRPGGR-NH_2

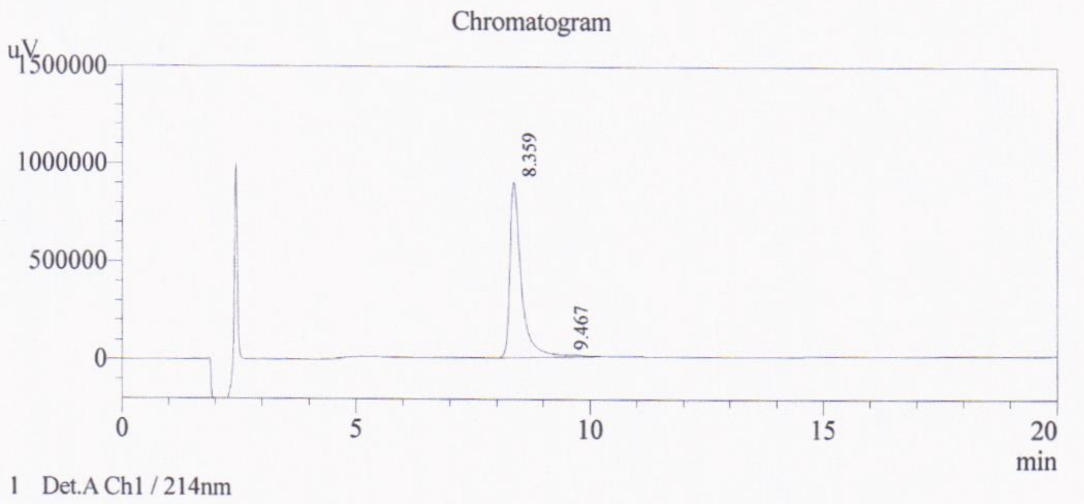

PeakTable

Detector A Ch1 214nm

Peak#	Ret. Time	Area	Height	Area %	Height %
1	8.359	15169053	893883	98.556	98.879
2	9.467	222310	10136	1.444	1.121
Total		15391363	904019	100.000	100.000

(o) VDKPPYLPRPRPIARPGGR-NH_2

Chromatogram

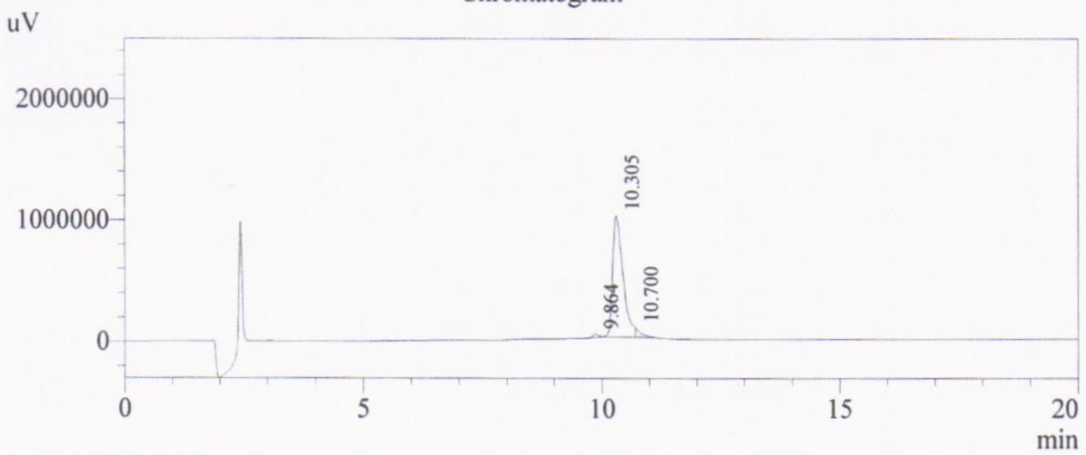

1 Det.A Ch1 / 214nm

PeakTable

Detector A Ch1 214nm

Peak#	Ret. Time	Area	Height	Area %	Height %
1	9.864	202835	23227	1.280	2.118
2	10.305	15096932	994358	95.263	90.672
3	10.700	547945	79068	3.458	7.210
Total		15847713	1096653	100.000	100.000

(p) VDKPPYLPRPRPIRAPGGR-NH_2

Chromatogram

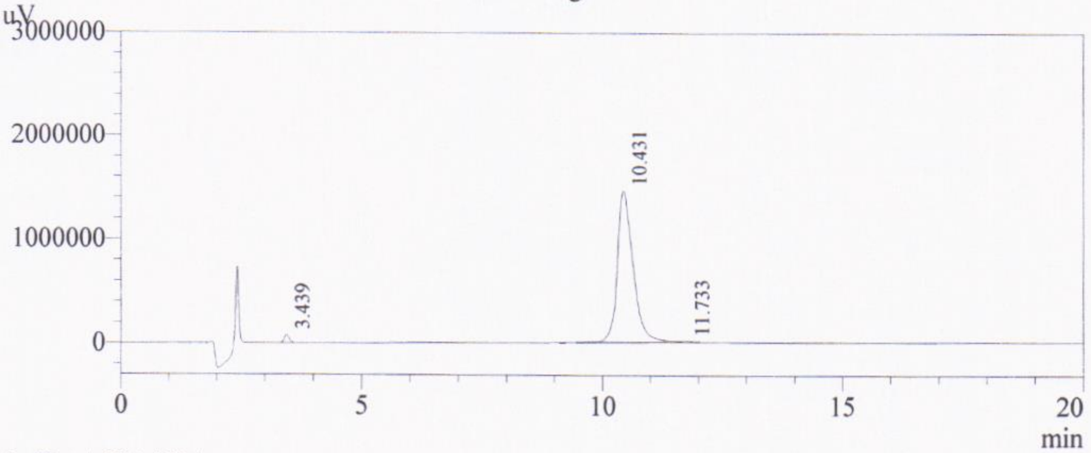

1 Det.A Ch1 / 214nm

PeakTable

Detector A Ch1 214nm

Peak#	Ret. Time	Area	Height	Area %	Height %
1	3.439	544382	76459	1.588	4.959
2	10.431	33546027	1453964	97.867	94.309
3	11.733	186778	11282	0.545	0.732
Total		34277186	1541704	100.000	100.000

(q) VDKPPYLPRPRPIRRAGGR-NH_2

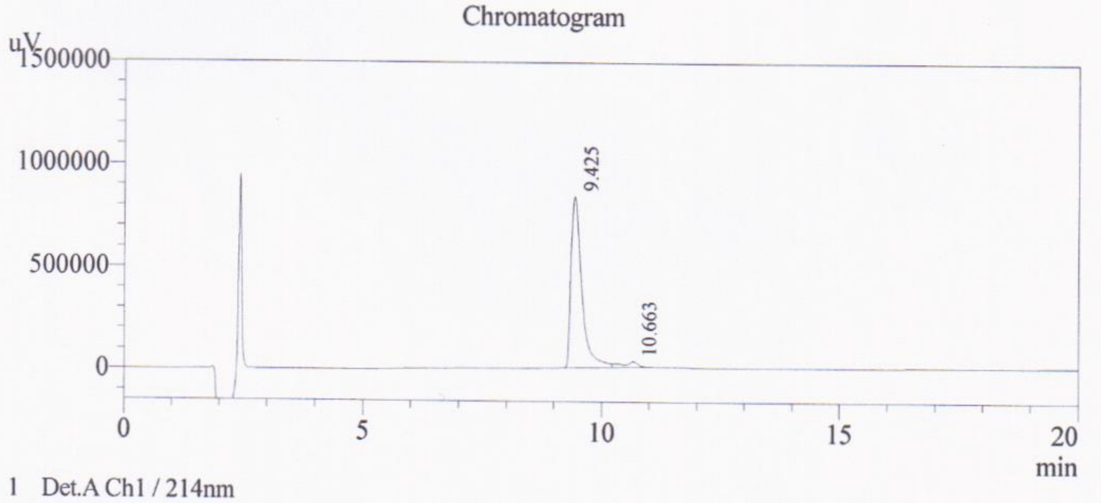

PeakTable

Detector A Ch1 214nm

Peak#	Ret. Time	Area	Height	Area %	Height %
1	9.425	13359808	835937	95.061	96.776
2	10.663	694113	27846	4.939	3.224
Total		14053920	863783	100.000	100.000

(r) VDKPPYLPRPRPIRRPAGR-NH_2

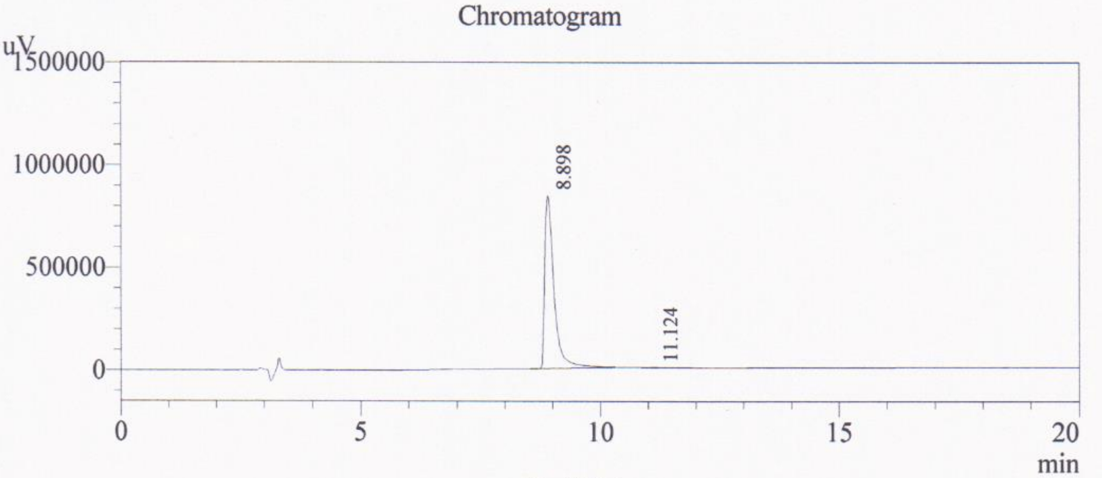

PeakTable

Detector A Ch1 214nm

Peak#	Ret. Time	Area	Height	Area %	Height %
1	8.898	11279457	842095	99.941	99.871
2	11.124	6647	1088	0.059	0.129
Total		11286104	843183	100.000	100.000

(s) VDKPPYLPRPRPIRRPGAR-NH_2

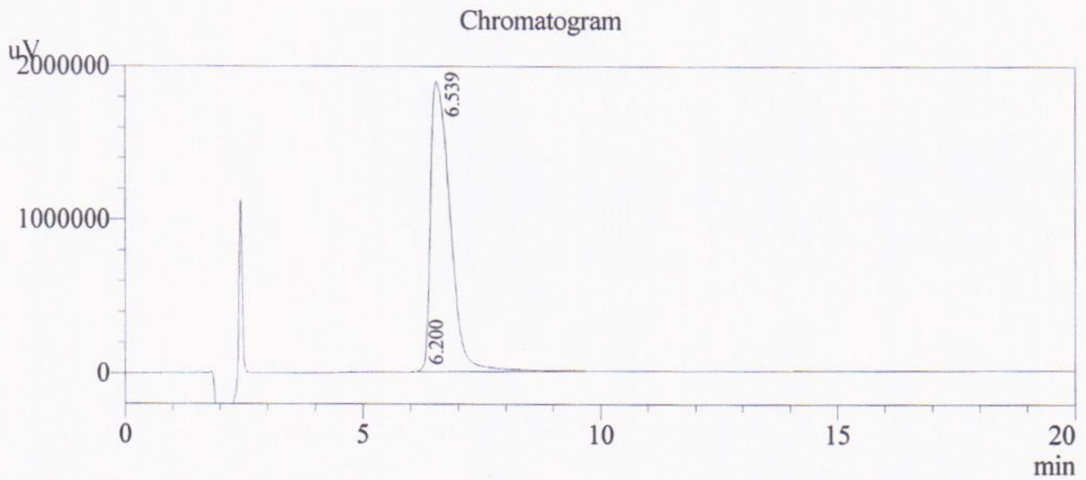

1 Det.A Ch1 / 214nm

PeakTable

Detector A Ch1 214nm

Peak#	Ret. Time	Area	Height	Area %	Height %
1	6.200	33237	10798	0.061	0.567
2	6.539	54024864	1892206	99.939	99.433
Total		54058101	1903004	100.000	100.000

(t) VDKPPYLPRPRPIRRPGGA-NH_2

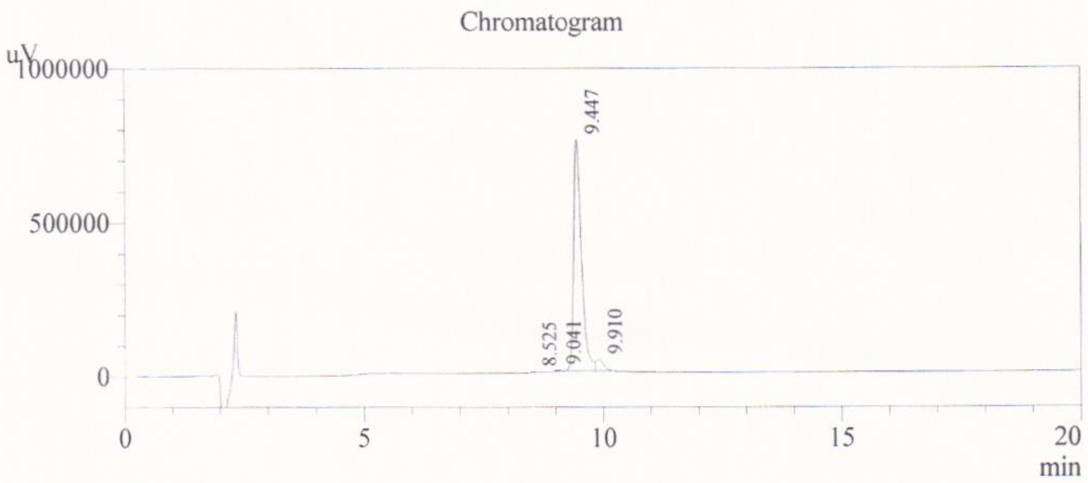

1 Det.A Ch1 / 214nm

PeakTable

Detector A Ch1 214nm

Peak#	Ret. Time	Area	Height	Area %	Height %
1	8.525	9802	1832	0.108	0.231
2	9.041	28384	4880	0.314	0.615
3	9.447	8593997	749310	95.095	94.374
4	9.910	405092	37956	4.482	4.781
Total		9037274	793977	100.000	100.000

(u) VDkPPYLPrPrPIrrPGGr-NH_2 (OM19D)

Figure S2. The HPLC chromatographic profiles of Peptides.

Table S1. Antimicrobial susceptibility test of clinical isolated strains used in this study (MIC, μg/mL).

	Antibiotics									
	**Sulfafurazole**	**Florfenicol**	**PolymixinB**	**Ceftriaxone**	**Gentamicin**	**Ciprofloxacin**	**Azithromycin**	**Tetracycline**	**Rifampicin**	**methicillin**
Escherichia Coli SN5	>512/R	4/S	0.5/S[1]	16/R[1]	<0.03/S	16/R	4/S	64/R	1/S	64/R
Escherichia Coli S1N1	512/R	2/S	0.5/S	16/R	<0.03/S	8/R	4/S	32/R	2/S	128/R
Escherichia Coli w136	512/R	128/R	8/R	0.06/S	8/I[1]	8/R	2/S	128/R	64/R	-
Escherichia Coli w122	512/R	128/R	0.5/S	32/R	1/S	<0.03/S	4/S	32/R	4/S	64/R
Escherichia Coli w123	512/R	128/R	0.5/S	32/R	1/S	<0.03/S	4/S	32/R	4/S	128/R
Escherichia Coli w124	512/R	128/R	0.5/S	4/R	8/I	4/R	4/S	128/R	4/S	-
Escherichia Coli QY	512/R	128/R	1/S	64/R	>512/R	64/R	8/S	256/R	4/S	-
Shigella flexneri QY1	>512/R	2/S	0.5/S	4/R	1/S	8/R	64/R	32/R	2/S	32/R
Methicillin-resistant Staphylococcus aureus HP	-	-	-	-	16/R	1/S	1/S	32/R	8/R	128/R

[1]Antimicrobial susceptibility test results were determined according to CLSI 2015 guidelines, where "R" stands for drug resistance, "I" stands for intermediate, "S" stands for susceptible.

Table S2. PCR primer sequences.

Gene	Sequence
16S	Forward primer: 5'-AGAGTTTGATCCTGGCTCAG-3'<br>Reverse primer: 5'-GGTTACCTTGTTACGACTT-3'
